# Reducing Friction on Skin at Risk: The Use of 3M^™^ Cavilon^™^ No Sting Barrier Film

**DOI:** 10.1089/wound.2015.0628

**Published:** 2015-12-01

**Authors:** Stéphanie F. Bernatchez, Golie E. Mengistu, Bruce P. Ekholm, Shilpi Sanghi, Steven D. Theiss

**Affiliations:** ^1^ Critical and Chronic Care Solutions Division, 3M, St. Paul, Minnesota.; ^2^ Corporate Research Materials Laboratory, 3M, St. Paul, Minnesota.

## Abstract

**Objective:** To compare the coefficient of friction (CoF) of skin against fabric when the skin is covered with a liquid barrier film versus a silicone dressing, relative to a bare skin baseline.

**Approach:** A laboratory instrument allowing the measurement of friction between two surfaces was used to compare the CoF between a fabric representing bed linen (100% cotton) and the skin of two laboratory operators, either bare (dry or hydrated) or covered with a liquid barrier film or a silicone dressing.

**Results:** The CoF of hydrated skin was over twice the value found for dry skin. The liquid barrier film product reduced the CoF of hydrated skin to a greater extent than the silicone dressing.

**Innovation and Conclusion:** Silicone dressings have recently been promoted to help prevent pressure ulcers. Published data have shown that their CoF is lower than other dressings, but the data were not compared to bare skin. We found that a liquid barrier film provided a greater reduction in the CoF of skin against linen than a silicone dressing. In the context of preventative use (*e.g.*, application on intact skin) to reduce the risk of pressure ulcers, applying a liquid barrier film may reduce friction better than a silicone dressing.

**Figure:**
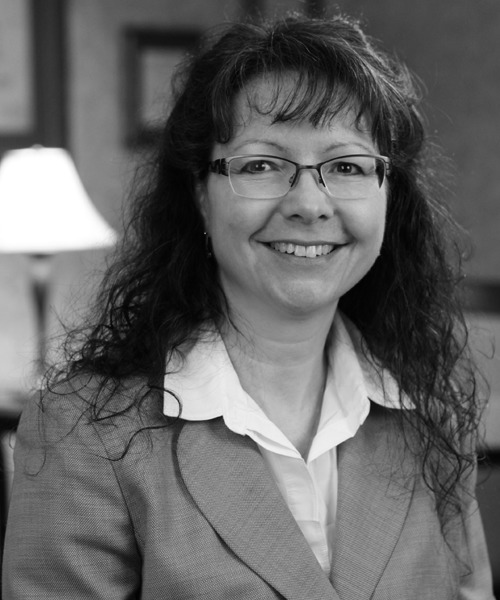
**Stéphanie F. Bernatchez, PhD**

## Introduction

The skin constitutes the protective layer of the body and provides an important anatomical barrier to pathogens, environmental threats, and mechanical injury. Various conditions can damage the skin and create a breach of its integrity, causing pain and increasing the risk of infection. Examples of damage-inducing conditions frequently occurring in the aging population are incontinence, lack of mobility leading to pressure points, and/or shear and friction during repositioning, as well as the frequent use of medical adhesive to secure various devices. Elderly people are particularly at risk for skin damage under those circumstances given the changes that occur in skin with the aging process.^1–4^ An international consensus document was recently published addressing pressure ulcer prevention and briefly mentions to “consider using film dressings to protect body areas at risk for friction injury or risk of injury from tape.”^5^

Several commercially available products target pressure ulcer risk reduction, such as pressure redistribution mattresses and dressings.^6^ Specialty fabrics have also been developed to reduce friction with skin compared to standard hospital linen.^7–9^ The current trend is to search for topical products that could reduce friction forces on the skin and represent a relatively inexpensive approach to help prevent the development of pressure ulcers and/or superficial friction injury.

Friction is one of the four mechanical factors contributing to pressure ulcer development, along with pressure, shear, and moisture. The effect of each of these factors toward pressure ulcer development varies, but it is generally accepted that minimizing or eliminating friction between the body surface and the support surface will reduce shear and thereby reduce the risk of pressure ulcer development. Friction can also create superficial skin injuries independent of pressure and shear. This is problematic in any location where skin surfaces rub together or against bed linen or clothing.

Friction can be quantified by measuring the coefficient of friction (CoF) between surfaces. This CoF is generally defined as a constant μ in the equation F=μW, where F is the frictional force and W is the load applied to the materials. In the case of viscoelastic materials, such as skin, however, μ is not always constant, particularly at low loads.^10^ It is known that moist skin has a higher CoF than dry skin^11^ and that this makes it more susceptible to friction blisters.^12^ Veijgen *et al.* have studied the correlation between CoF and skin temperature, skin hydration, and age of the subject in different body locations and presented multivariable models for the static and dynamic CoFs.^13^ The static CoF is calculated with the friction force that is required to initiate the movement between the skin and contact cylinder at a certain normal load, while the dynamic CoF is calculated with the friction force that is needed to retain the movement once initiated. The authors concluded that the static and dynamic CoFs were strongly correlated. Other studies on human volunteers determined that the CoF and other skin properties vary with age, gender, and body site.^14–16^

A relatively recent development is the promotion of silicone dressings to reduce the risk of pressure ulcer development. This is based on the concept that such dressings can reduce the shear stress and friction reaching the skin of the patients at risk. Call *et al.* have published a study comparing the CoF of various dressings.^17^ They found that different dressings vary significantly from each other and that the size of the dressing also plays a role in the outcome of the friction test. This work, however, has not documented the baseline CoF of bare skin in comparison to the dressings tested. Some clinical studies have also been published on this topic, but they either have found no statistical difference between the intervention and control groups^18^ or the study involved several interventions, and it is not possible to isolate the effect of the silicone dressing alone on the improved outcome.^19,20^

## Clinical Problem Addressed

Pressure ulcer prevention is the clinical problem related to this article. Our experimental approach measured the CoF, which is only one component of this complex problem. Liquid barrier films have been used for friction reduction for many years, but data supporting this practice are anecdotal. We wondered how a liquid barrier film would compare to a dressing for its effect on the CoF of skin. The objective of this study is to describe laboratory results measuring the friction of skin against fabric and to report the friction properties of a liquid barrier film (3M™ Cavilon™ No Sting Barrier Film) compared to a silicone dressing (Mepilex^®^ Foam Dressing from Mölnlycke).

## Materials and Methods

### Products tested

• 3M Cavilon No Sting Barrier Film (1 mL swab, REF No. 3343, Lot 2013-12 BA; 3M, St. Paul, MN).
• Mepilex Border Foam Dressing (Lot No. 10396706, 2013-09 and Lot No. 11122362, 2014-03; Mölnlycke Health Care, Norcross, GA).

### Instrument

**Figure 1.**
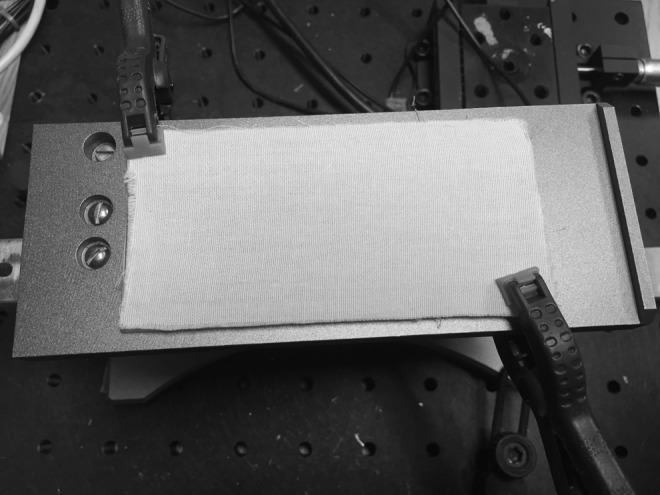
Test equipment to measure friction between surfaces. The fabric used to model standard hospital linen is laminated on the glass plate of the test equipment.

**Figure 2.**
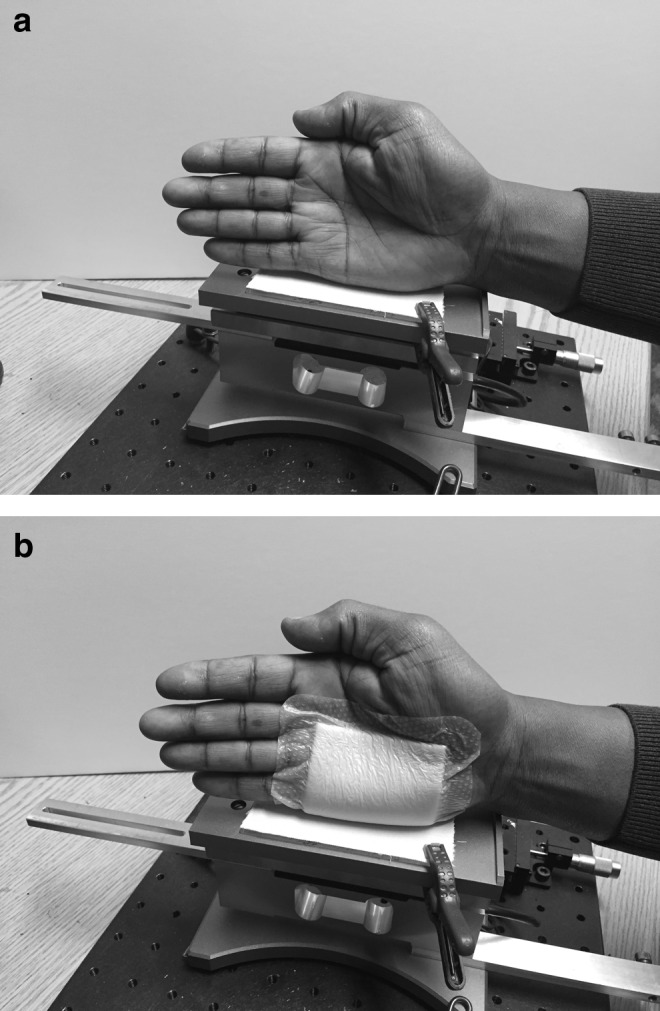
Test equipment in use by operator. **(a)** Bare skin. **(b)** Skin with test dressing (example of test product).

An instrument (ForceBoard™; Industrial Dynamics Sweden AB, Jarfalla, Sweden) allowing the measurement of friction between two surfaces was used to compare the CoF between a fabric representing bed linen (100% cotton) and the skin of two laboratory operators, either bare (dry or hydrated) or covered with the test products. The fabric was laminated onto a flat sliding glass (5.75 in by 2.25 in) using a double-sided adhesive to avoid wrinkling when rubbing against the test material, as seen in Figure 1. A new piece of fabric was used for each replicate of each sample tested to avoid changing the fabric's geometry (texture), since texture grooves are a major frictional contributor and might lead to a surface topography effect. The ForceBoard instrument is based on two orthogonal load cells (consisting of resistive strain gauges in a Wheatstone bridge configuration), where a test material is drawn across the surface of the sample of interest, as shown in Figure 2. The normal and tangential mechanical loads during a friction test are converted into voltage signals that are amplified and proportional to the load. These forces are sampled at 10 Hz using DAQFactory software (AzeoTech, Ashland, OR).

### Assay conditions

The hands of two different laboratory operators were washed with soap and water and dried completely before the test (for control, dry skin). Three replicate runs were taken, changing the fabric for each run. Each run consisted in a series of 12 sequential horizontal strokes against the whole length of the glass plate. Once all data were collected, the data of the three replicate runs were pooled and averaged.

In addition, the test was also performed using the same method after soaking the hand of the operators in water at room temperature for 5 min and lightly blotting it dry before the test (for control, hydrated skin). This induced a higher level of skin hydration, a parameter known to affect the CoF of skin. The intent was to more closely simulate the skin condition found in the sacral area of patients in hospital beds.

To test the liquid barrier film, the product was applied on the side of the hand (hydrated skin condition) and rubbed against the fabric laminated to the glass plate of the instrument to mimic the friction that occurs between a patient and the hospital bed linen. For the silicone dressing, the product was applied on the side of the hand using the dry skin condition to not affect the adhesion of the product. Each material was tested using three to six replicate runs.

### Measurement of the CoF

The instrument reading provided the time, the horizontal force (friction), the vertical force (how hard the hand pushed on the plate), and the quotient of horizontal/vertical, or CoF. The data were sorted by vertical force and all values below 0.1 N were eliminated (to remove the data points between the strokes within each run). The CoF of the remaining data points was averaged for each test material.

**Figure 3.**
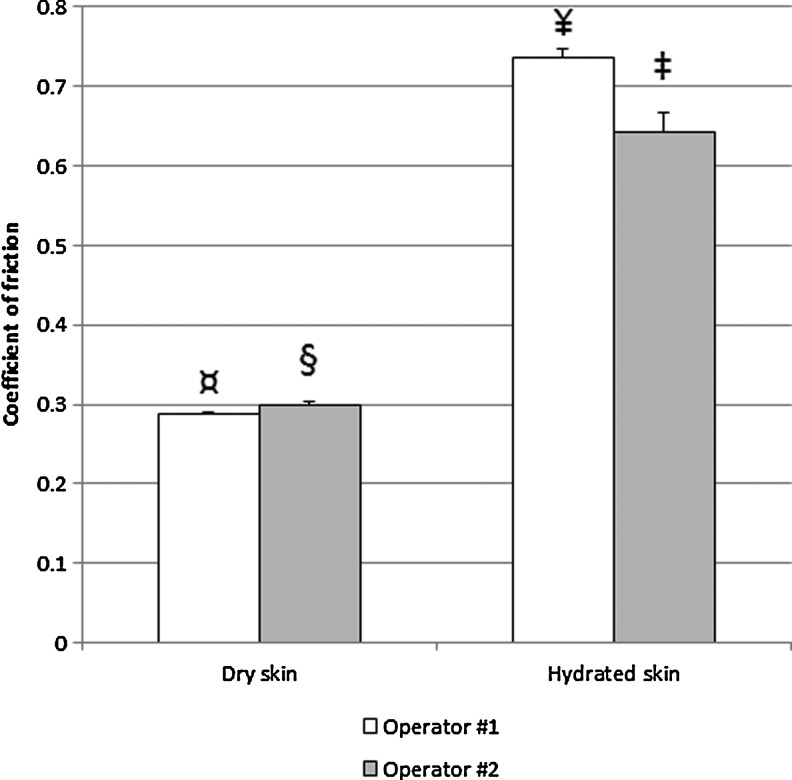
CoF measured on dry and hydrated skin in two laboratory operators. The standard error of the mean is displayed. The differences between operators (*p*<0.001 by Student's *t*-test) and skin conditions (*p*<0.001 by ANOVA) were significant, as illustrated with a different symbol for each bar. ANOVA, analysis of variance; CoF, coefficient of friction.

**Figure 4.**
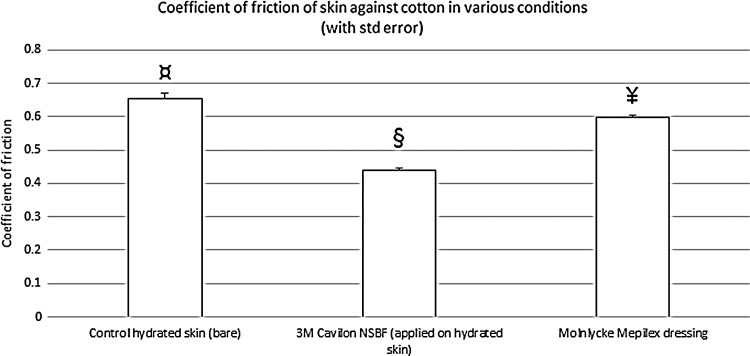
CoF for the test products compared to bare skin (control). The standard error of the mean is displayed. All means were significantly different from each other (*p*<0.001 by ANOVA), as illustrated with a different symbol for each bar. Using Tukey's method, 3M Cavilon NSBF was significantly lower than both Mepilex and control; Mepilex was significantly lower than control.

The vertical force applied was not constant since it was driven by the operator (as opposed to a set point on a piece of equipment). In fact, the operator applied a gradual increase in the force pushing against the plate during each stroke to generate data with a range of forces. For this reason and to obtain information on the effect of the vertical force on the CoF, the data were binned for each test material to average results obtained with the following vertical force ranges: 0.1–4, 4.1–6, 6.1–10, 10.1–15, and 15.1–25 N. As expected, the variability was larger at low vertical force, but the averages for the different bins were not practically different (data not shown). Therefore, the average CoF for all data points with a vertical force above 0.1 N was chosen to display the data, as shown in Figures 3 and 4.

### Statistical analysis

The operator-to-operator data were compared using an analysis of variance (ANOVA) with operator, skin condition, and interaction as factors in the model. Least squares estimates of the means for hydrated and dry skin were obtained from this model. Because the interaction was significant (*p*<0.001), the operators were compared using a Student's *t*-test.

The CoF for the test materials was compared using a one-way ANOVA. Significance was defined as the *F*-test for having a *p*-value<0.05. If significant, multiple comparisons were done using Tukey's method to control the experimentwise Type I error.

## Results

### Effect of moisture on skin friction

The CoF was measured in triplicate for two different operators, for dry skin and for hydrated skin. A clear difference was observed between those conditions, with the CoF being approximately twice as high for hydrated skin, as shown in Figure 3. These data show the baseline friction of bare skin against the fabric sample. Differences were also seen between operators, with a higher variability observed for hydrated skin. For dry skin, operator 2 was 3.7% higher than operator 1 (*p*<0.001 by Student's *t*-test). For hydrated skin, operator 2 was 12.8% lower than operator 1 (*p*<0.001 by Student's *t*-test). The overall CoF (pooled operators) of hydrated skin was 134.7% higher than the CoF of dry skin. This difference being largely superior to the variability between operators, we deemed adequate to use the hydrated skin average of both operators to later compare the CoF of the test products against the same type of fabric, as shown in Figure 4.

### CoFs for test products

Figure 4 illustrates the friction measured when the skin of one of the operators was covered with each of the test products to run the assay. Both test products significantly reduced the CoF of skin against fabric (*p*<0.001). 3M Cavilon No Sting Barrier Film provided a statistically significant greater reduction than the Mepilex dressing. The CoF measured with 3M Cavilon No Sting Barrier Film was 32.8% lower than that measured on control skin, which is well outside of the variability between operators. The CoF measured with the Mepilex dressing was only 8.6% lower than that measured on control skin, which is within the range of the variability between operators.

## Discussion

The frictional force experienced by skin rubbing against fabric is influenced by the load applied to it. This can depend on the body part involved and its weight (*e.g.*, the sacrum of a person in an elevated supine position in bed exerts more force against the bed than an arm rubbing against a chair armrest), and also on the overall weight of the patient. In addition, the condition of the skin (aged vs. young, overhydrated vs. dry) influences how it can tolerate friction before experiencing damage.

Our results show that the CoF of hydrated skin was roughly twice that of dry skin. This rise in the CoF with increasing skin hydration is consistent with previously published observations.^11^ The CoFs measured in this study lie in the same range as other results reported in the literature.^15^ In our test system, we chose a gradual increase in the force applied by the hand of the operator pushing against the plate. This allowed us to collect data at various forces. We observed that the variability in the CoFs was higher at lower forces, which was expected from previously published work.^8^ For our range of forces, however, the CoF remained rather constant. In principle, the CoF should be a constant, but variations are seen for viscoelastic materials such as skin. It is possible that, however, the test site we chose (the side of the hand) has less viscoelasticity than other body sites, such as the sacral area, since the underlying tissues do not have as much depth.

The 3M Cavilon No Sting Barrier Film provided the greatest reduction in CoF against fabric. The Mepilex dressing also reduced the CoF compared to bare hydrated skin, but to a lesser extent, which was within the variability observed between operators. In the article by Call *et al.*,^17^ several dressings were tested (including Mepilex), but CoF data on bare skin were not presented. These authors used the BS 3424-10:1987 method,^21^ with modifications. This test method is in principle similar to ours, but the materials are tested against a control fabric (melton cloth) and no skin reference is used. We believe that our method, using skin on a human operator, provides a model with better clinical relevance. Limitations of our study are that it is still a bench model; we used the skin on the side of the hand and soaked it for 5 min to increase its hydration and this may not represent the exact condition of sacral skin from bedridden patients; and we collected data with a small number of operators and products. Nevertheless, we saw statistically significant differences.

A dressing obviously needs to be applied if there is a pressure ulcer that requires protection. However, when it comes to prevention, the possible benefits of a preventive dressing must be weighed against other options that may be less expensive and/or less labor intensive. Even though the Mepilex dressing was the one with the lowest CoF in the Call study,^17^ our data show that this dressing displays a higher CoF than skin protected by 3M Cavilon No Sting Barrier Film. Another benefit from the use of skin barriers is that there is no need to remove this product, since it gradually sheds away with the skin desquamation process. The product can be reapplied as needed without any removal step risking to damage the fragile skin being treated. Dressing changes, on the other hand, present the risk of additional trauma to the skin.

In this bench model, 3M Cavilon No Sting Barrier Film provided a significantly greater reduction in the CoF than the Mepilex dressing. In the context of preventative use (*e.g.*, application on unwounded skin) to reduce the risk of pressure ulcers, applying a liquid barrier film may be more effective than a dressing to reduce the friction between skin and linen.

## Innovation

Pressure ulcer prevention requires addressing several factors, including pressure, shear, moisture, friction, and patient mobility. A current clinical approach advocates the use of silicone dressings as one of the several interventions,^20^ and friction reduction has been proposed as a possible mode of action. Our work using a bench model shows that a barrier film provides a greater reduction of the CoF against fabric than a silicone dressing. The potential innovation in this approach is the possibility to use a simpler, less expensive product to achieve friction reduction in patients at risk for the development of pressure ulcers.

## About the Authors

**Stéphanie F. Bernatchez, PhD,** received her PhD in Interdisciplinary Sciences from the University of Geneva, Switzerland. She currently resides in Minnesota and works for 3M. **Golie E. Mengistu, MS,** received his MS in Regulatory Science from the University of St. Thomas, School of Engineering, St. Paul, MN. He currently resides in Minnesota and works for 3M. **Bruce Ekholm, MS,** received his MS in Statistics from the University of Minnesota and currently works at the 3M Company in Minnesota. **Shilpi Sanghi, PhD,** received her PhD in Polymer Science and Engineering from the University of Massachusetts Amherst. She worked for 3M for 3 years and currently resides in San Francisco. **Steven D. Theiss, PhD,** received his PhD in Applied Physics from the Harvard University and currently works for 3M in Minnesota. His work at 3M has included a wide variety of problems in Materials Science, including studies of tactile friction.

## Abbreviations and Acronyms

ANOVA: analysis of variance
CoF: coefficient of friction
